# 3D‐printable lung phantom for distal falloff verification of proton Bragg peak

**DOI:** 10.1002/acm2.12706

**Published:** 2019-09-20

**Authors:** Junichi Koketsu, Hiroaki Kumada, Kenta Takada, Hideyuki Takei, Yutaro Mori, Satoshi Kamizawa, Yuchao Hu, Hideyuki Sakurai, Takeji Sakae

**Affiliations:** ^1^ Proton Medical Research Center University of Tsukuba Hospital Tsukuba Ibaraki Japan; ^2^ Faculty of Medicine University of Tsukuba Tsukuba Ibaraki Japan; ^3^ Department of Radiological Technology Gunma Prefectural College of Health Sciences Maebashi Gunma Japan

**Keywords:** 3D printer, lung phantom, measurement, Monte Carlo simulation, proton therapy, quality assurance

## Abstract

In proton therapy, the Bragg peak of a proton beam reportedly deteriorates when passing though heterogeneous structures such as human lungs. Previous studies have used heterogeneous random voxel phantoms, in which soft tissues and air are randomly allotted to render the phantoms the same density as human lungs, for conducting Monte Carlo (MC) simulations. However, measurements of these phantoms are complicated owing to their difficult‐to‐manufacture shape. In the present study, we used Voronoi tessellation to design a phantom that can be manufactured, and prepared a Voronoi lung phantom for which both measurement and MC calculations are possible. Our aim was to evaluate the effectiveness of this phantom as a new lung phantom for investigating proton beam Bragg peak deterioration. For this purpose, we measured and calculated the percentage depth dose and the distal falloff widths (DFW) passing through the phantom. For the 155 MeV beam, the measured and calculated DFW values with the Voronoi lung phantom were 0.40 and 0.39 cm, respectively. For the 200 MeV beam, the measured and calculated DFW values with the Voronoi lung phantom were both 0.48 cm. Our results indicate that both the measurements and MC calculations exhibited high reproducibility with plastinated lung sample from human body in previous studies. We found that better results were obtained using the Voronoi lung phantom than using other previous phantoms. The designed phantom may contribute significantly to the improvement of measurement precision. This study suggests that the Voronoi lung phantom is useful for simulating the effects of the heterogeneous structure of lungs on proton beam deterioration.

## 1 INTRODUCTION

The Bragg peak in the radiation dose distribution in proton therapy allows for better dose distribution compared to other radiation treatments,^1^, ^2^, ^3^ so proton therapy is one of the important options for cancer treatment. However, researchers have reported that passage of the proton beam through heterogenous regions, such as the human lung, causes the Bragg peak to be amplified, deteriorating the distal falloff.^4^, ^5^, ^6^ This amplification may inadvertently cause an increase in the radiation dose to normal tissues or a decrease in the radiation dose to cancer cells.

In previous research, a heterogeneous random voxel calculation model, in which soft tissue and air are randomly allotted to equal the average density of lungs, we call this porous walled structure phantom or random voxel phantom, was used to investigate beam deterioration using Monte Carlo (MC) simulation.^7^ The researchers fabricated a three‐dimensional (3D) printed porous walled structure phantom to verify the calculation results and the measurement. To print the 3D phantom from the data, a different support material from the main unit must be used to compile the 3D structure, and it must be removed at the end. However, because the model is randomly composed, in case of porous walled structure phantom, some spaces may be created where the support material cannot be removed. As a result, the randomness of the physical phantom that can be fabricated with a 3D printer, is limited.

Therefore, we devised a 3D printable lung phantom that is not limited in randomness and can be employed to perform not only calculations but also advanced measurements, by using a mathematical technique called Voronoi tessellation.

The purpose of this research was to verify the effectiveness of the new lung phantom developed using Voronoi tessellation, by comparing the Bragg peak deterioration of the proton beam with that observed in previous research, using both measurements and MC calculations.

## 2 MATERIALS AND METHODS

### 2.1 Voronoi lung phantom

#### 2.1.1 Voronoi tessellation

**Figure 1:**
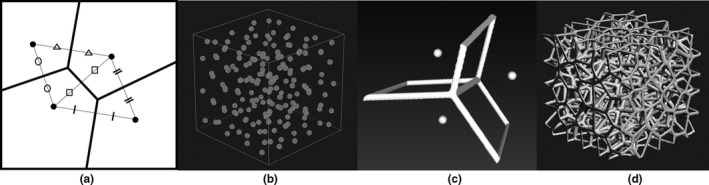
Simple illustration of Voronoi tessellation. Generating points are randomly placed within a square (a). Randomly generating points so as to be the centroidal Voronoi tessellation in three dimensions (b). Structuring the Voronoi tessellation lines in three dimensions (c). Formation of a porous branch structure by repeating the same procedure as in (c) for all the generated points (d).

We focused on using Voronoi tessellation to print a lung structure. Voronoi tessellation is a mathematical technique, in which a perpendicular straight line is made at the midpoint of the line between neighboring points, dividing an area into the regions that are closest to each point [Fig. 1(a)]. In this study, we used centroidal Voronoi tessellation (CVT),^8^, ^9^ where the generating point in each region is also the center of mass. Because the gaps between the generating points are uniform, we used this tessellation with the aim of decreasing directional dependence related to the structure.

#### 2.1.2 Lung phantom using Voronoi tessellation

In this study, MeshLab (JS16.03),^10^ an open‐source modeling program for 3D model design was used. The generating points were placed in a 4 cm × 4 cm × 4 cm cube using the Poisson disk distribution^11^ and Lloyd's algorithms,^8^, ^9^ installed as functions of MeshLab [Fig. 1(b)]. A Voronoi tessellation was created using these points, and the basic shape of the phantom was developed by structuralizing the Voronoi tessellation lines [Fig. 1(c)]. It was possible to make a porous branch structure using the structuralized dividing lines [Fig. 1(d)]. A porous branch structure has the advantage of easy removal of the support material. This means that it is possible to create a phantom with less design error than conventional porous walled structures. Afterward, based on studies of human lungs,^12^, ^13^ we developed a Voronoi lung phantom with a density of 0.237 g/cm^3^ and branch diameters of 0.4–0.8 mm to approximate an average adult lung. The joint part of the branches was thicker and about 1.6 mm in diameter at maximum. The Voronoi lung phantom was fabricated with an inkjet 3D printer AGILISTA‐3100 (KEYENCE) in the Medical Workshop and the Open Facility Network Office at the Research Facility Center for Science and Technology of the University of Tsukuba. Transparent acrylic urethane resin (density: 1.11 g/cm^3^) was used to form the model. In order to verify that the support material was removed, the design density was compared with the density of the actual phantom.

### 2.2 Comparison parameters

We compared the Voronoi lung phantom's distal falloff widths (DFW) and peak values obtained from the measurements and MC calculations with those of a previously studied plastinated human lung sample.^7^

The DFW is defined as the distance required for the radiation dose distribution to fall from 80% to 20% after a peak. The peak value is the value of the peak, taking a pristine peak to be standard, i.e., normalized with the maximum dose of the pristine peak.

We measured and simulated the percentage depth dose (PDD), from which the DFW and peak values were calculated.

### 2.3 Measurement setup

Measurements were conducted with a passive beam, using the double scattering technique, at the University of Tsukuba Hospital Proton Medical Research Center (PMRC). We evaluated the difference in the PDD with and without the Voronoi lung phantom. We used 155 and 200 MeV beams, which are often used in the treatment of the lungs.

**Figure 2:**
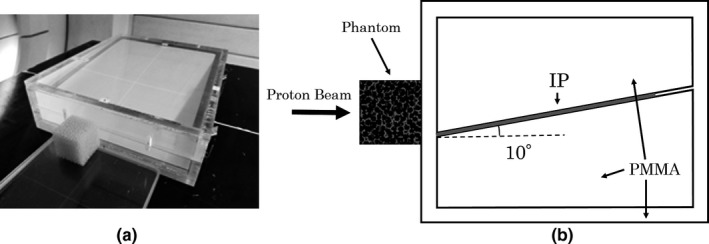
Photograph of the imaging plate set in a PMMA container and the three‐dimensional printed phantom in place (a). Diagram of the experimental system (b).

The instrument for measuring PDD was an imaging plate (IP).^14^ The experimental system involved the Voronoi lung phantom being placed in front of an IP detector and irradiated with proton beams at 155 and 200 MeV [Fig. 2(a)].

The method to measure the PDD with an IP is described in the literature.^15^ A polymethyl methacrylate (PMMA) (1.17 g/cm^3^) container was used for this purpose. The IP was inserted at an angle of 10°. The oblique incidence changes the distance the protons pass through the sensitive layer of the IP, which also changes the signal intensity. Therefore, in the previous study,^15^ the change in signal intensity was used to determine the optimum angle for compensating the LET dependency of IP. By measuring with IP at 10°, the measurement result in the distal falloff part was in agreement with that using parallel plate ionization chamber in water. For details on this technique, please refer to the literature.^15^

The IP that was used was a BAS‐MS (FUJI FILM), the scanning device was an FLA‐7000 (FUJI FILM), and the analysis software was Multi Gauge (FUJI FILM). The resolution in the depth direction of measurement was 0.1 mm of the minimum reading size.

### 2.4 Monte Carlo simulation

The MC simulations reproduced the PMRC's double scattered beamline using the Particle and Heavy Ion Transport Code System (PHITS) ver3.02.^16^ In a previous study,^17^ for the PMRC beamline, the PDD of a 155 MeV proton beam was evaluated using Monte Carlo simulation, and the measured values were reproduced. In this study, Monte Carlo simulations were carried out by adapting that calculation system. The calculation system used the same geometry as used for the measurements [Fig. 2(b)], which was constructed in the PHITS. The PDD was calculated under the same conditions as the measurements. We calculated the PDD with or without the Voronoi lung phantom for the energy levels of 155 and 200 MeV.

**Figure 3:**
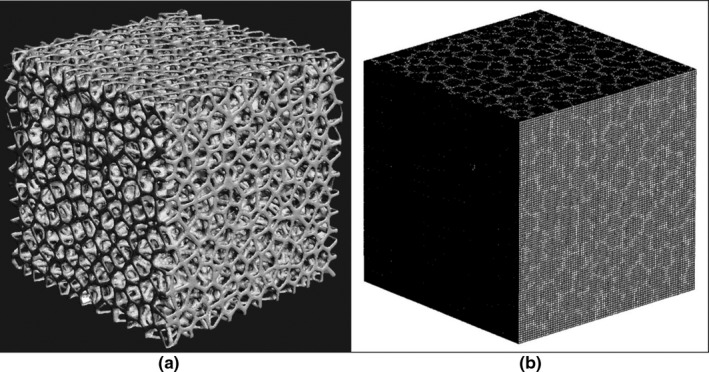
The created three‐dimensional data (a) were converted to voxel data (b) for Monte Carlo calculation by particle and heavy ion transport code system. Each voxel represented a volume of 0.4 mm × 0.4 mm × 0.4 mm. The structural parts that appear white are defined by acrylic urethane resin with a density of 1.11 g/cm^3^; the other parts are defined by air.

The calculation system was incorporated into the Voronoi lung phantom by dividing the 4 cm × 4 cm × 4 cm 3D data into a 100 × 100 × 100 grid, in which each voxel represented a volume of 0.4 mm × 0.4 mm × 0.4 mm (Fig. 3). It is a limitation that the MC modeled lung structure and the 3D printed lung phantom are not exactly the same because of the partial volume effects associated with the process of voxelization. The materials used were a transparent acrylic urethane resin with a density of 1.11 g/cm^3^ and PMMA with a density of 1.17 g/cm^3^.

The cutoff energies for protons and electrons were set to 1 and 0.1 MeV, respectively. The calculations for the simulation were performed with sufficient number of particles so that the statistical error for radiation doses was 1% or less up to a point 20% past the peak. The voxel size for calculating the depth dose was 0.2 mm because of trade‐off between statistical error and realistic computation time.

### 2.5 Comparison with tough lung phantom

We compared the effects of the heterogeneous random structure in the conventional and the Voronoi lung phantom. We used tough lung phantom (Kyotokagaku) as a conventional phantom. The tough lung phantom has a density of 0.33 g/cm^3^ (phenol formaldehyde resin) and is a uniform plate‐like phantom, with no heterogeneous structure. The experimental system was the same as in Fig. 2(b). In order to correct the difference in density with Voronoi lung phantom (0.237 g/cm^3^), we first confirmed that the results of the tough lung phantom with 3 cm thickness are matched in MC calculation and measurement. Second, a plate‐shaped virtual phantom made of the same material as tough lung phantom was made to have the same density as the Voronoi lung phantom in MC calculation. Then, a Voronoi lung phantom with a heterogeneous structure and a tough lung phantom without a heterogeneous structure were compared by MC calculation. We calculated the pristine peak's PDD for the energy levels of 155 and 200 MeV. The voxel size for calculating the depth dose was 0.2 mm.

### 2.6 Comparison with treatment planning system (TPS)

We compared our setup with the general treatment planning system (TPS) with pencil beam algorithm^18^ to investigate the effects of heterogeneous structure in near‐clinical conditions. VQA ver. 2.01 (Hitachi) was used as TPS. To perform the calculation for TPS, we took the CT of the Voronoi lung phantom we created. We obtained 0.977 mm × 0.977 mm × 0.625 mm (0.625 mm as slice thickness) with the best resolution in the range used in the clinic. CT was performed with an Optima 580w (GE). The experimental system was the same as in Fig. 2(b). We calculated the PDD for the energy levels of 155 and 200 MeV. We chose 30 mm as the spread‐out Bragg peak (SOBP) width, often used in the treatment of lungs, to compare with MC calculations. The TPS's voxel size for calculating the depth dose was 0.1 mm of the minimum reading size, while that of MC was 0.2 mm.

## 3 RESULTS

### 3.1 Voronoi lung phantom

**Figure 4:**
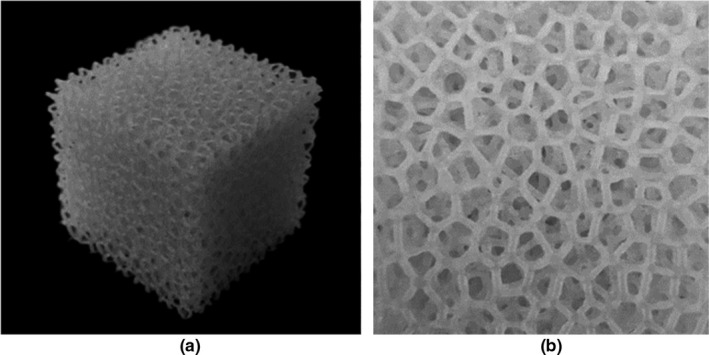
The actual printed phantom (a) and a cross section (b). The picture on the right shows a cross section of the phantom that demonstrates the features of the Voronoi tessellation. The phantom was 4 cm × 4 cm × 4 cm in size and had a density of 0.23 g/cm^3^.

A view and enlarged view of the constructed lung phantom are shown in Fig. 4. The measured density of this lung phantom was 0.237 g/cm^3^, which was consistent with the design. At more than three decimal places, the design density was 0.2371 g/cm^3^, and the actual phantom density was 0.2369 g/cm^3^. The error was < 0.1%. Hence, it can be said that the design was reproduced with high accuracy.

All measurements and calculations reported in this study were performed using this phantom.

### 3.2 Results of comparison between measurements and MC calculations

**Figure 5:**
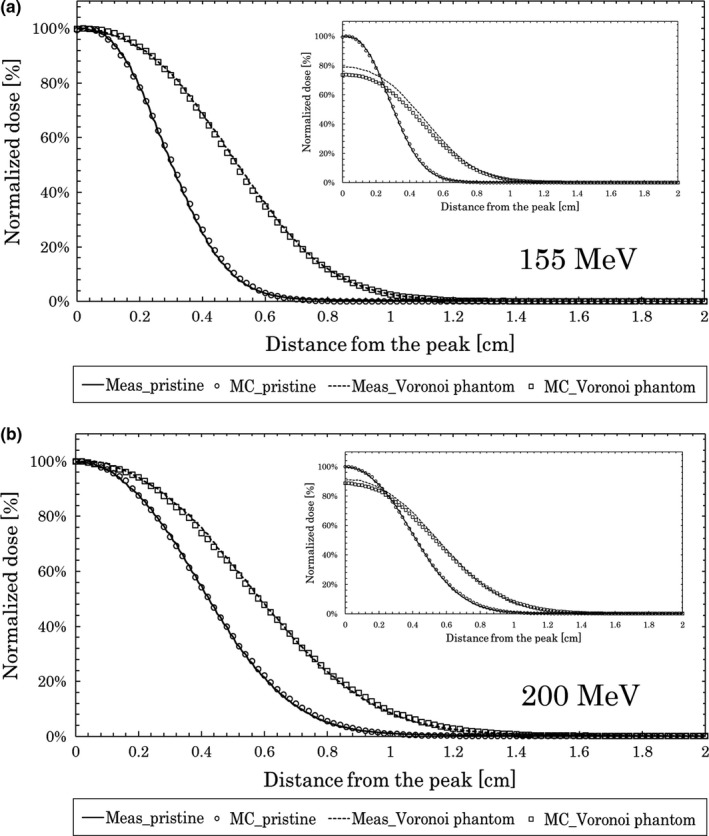
Measurements and Monte Carlo calculations for depth dose. Results at 155 MeV (a) and 200 MeV (b) with and without the Voronoi lung phantom.

The results for the pristine peak normalized by the maximum dose and the peak normalized by the maximum dose after passing through the Voronoi lung phantom are shown in Fig. 5. The graphs in the insets are the ones normalized with the maximum dose of the pristine peak. The horizontal axis is the length in the depth direction from the peak.

For the 155 MeV beam, the DFW values of the measurement and MC calculation were both 0.24 cm for the pristine peak. The measured and calculated DFW values with the Voronoi lung phantom were 0.40 and 0.39 cm, respectively, which are similar.

For the 200 MeV beam, the DFW values of the measurements and MC calculations were both 0.36 cm for the pristine peak. The measured and calculated DFW values with the Voronoi lung phantom were 0.48 cm.

The results for the DFW and peak value are shown in Table 1. From this table, we see that the measured and MC‐calculated Voronoi lung phantom peak values were 80% and 74%, respectively, for 155 MeV. For 200 MeV, the values were 91% and 89%, respectively.

**Table 1:** Measurement and Monte Carlo (MC) calculation results for distal falloff widths (DFW) and peak values.

Method	Setup	155 MeV		200 MeV	
		DFW (cm)	Peak value (%)	DFW (cm)	Peak value (%)
Measurement	Pristine peak	0.24	100	0.36	100
MC simulation	Pristine peak	0.24	100	0.36	100
Measurement	Pristine peak with Voronoi lung phantom	0.40	80	0.48	91
MC simulation	Pristine peak with Voronoi lung phantom	0.39	74	0.48	89
Measurement^a^	Pristine peak with plastinated lung sample^a^	0.35–0.40^a^	77–81^a^	0.48–0.51^a^	88^a^
Measurement^a^	Pristine peak with random voxel phantom^a^	0.51^a^	68^a^	0.61^a^	86^a^

a Referenced from Titt et al.^7^

Linear interpolation of the values from 140 to 200 MeV.

For the plastinated lung sample results from the previous study,^7^ the values measured at 140 MeV had a peak value of 74–78% and DFW of 0.31–0.35 cm, while those measured at 200 MeV had a peak value of 88% and DFW of 0.48–0.51 cm. The energy used in the present study (155 MeV) was different from the one used in the previous study (140 MeV), but linear interpolation of the values from the previous study indicates a DFW of 0.35–0.40 cm and a peak value of 77–81% at 155 MeV. These previous study results and the present study's results at 155 MeV were sufficiently consistent with the measured and calculated values for DFW. The peak values were also consistent with the measured and calculated values, within a maximum 5% error, compared to the previous study results. The results at 200 MeV were also sufficiently consistent with the measured and calculated values for DFW, and the peak values were also consistent with the measured and calculated values, within a maximum 3% error, compared to the previous study results. The measurement results of random voxel phantom with porous walled structure used in the previous research^7^ were: DFW of 0.51 cm, peak value of 68% for 155 MeV (linear interpolation at 140 MeV and 200 MeV listed^7^) and 0.61 cm, 86% for 200 MeV. In comparison with this result, it can be seen that the measurement of Voronoi phantom is closer to the result of plastinated lung sample.

Based on these results, the Voronoi lung phantom was judged to be suitable to simulate a plastinated lung sample made from the human body.

### 3.3 Comparison with tough lung phantom

**Figure 6:**
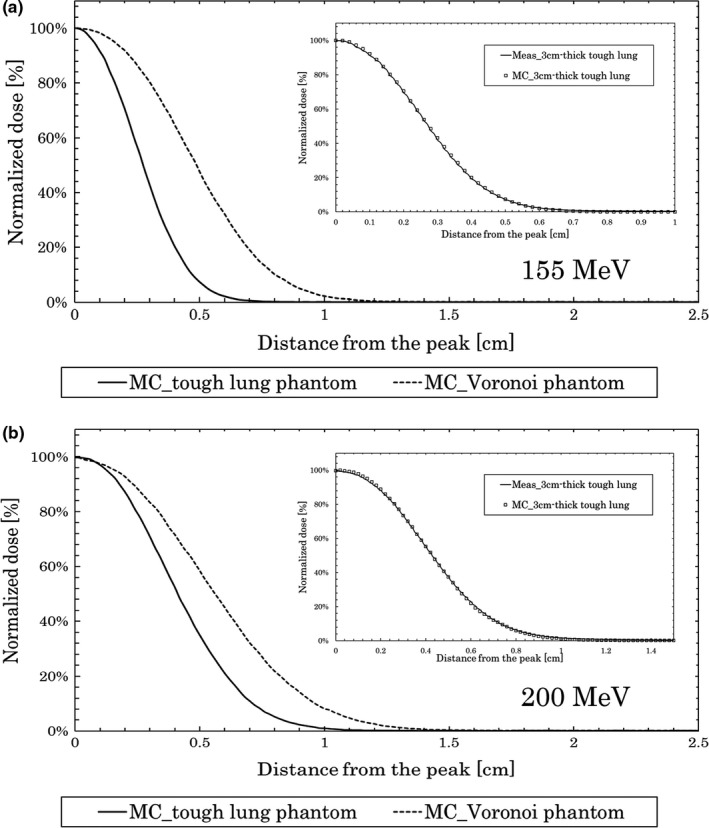
Monte Carlo calculations for depth dose. Results at 155 MeV (a) and 200 MeV (b) with the Voronoi lung phantom and with tough lung phantom.

Figure 6 shows the results of MC calculations for 155 MeV (a) and 200 MeV (b) when passing through the Voronoi lung phantom and tough lung phantom. The vertical axis of the graph was normalized by the maximum dose. The horizontal axis is the length in the depth direction from the peak. In the graphs in the inset, the actual measurement value and MC calculation value, when passing through the 3 cm‐thick tough lung phantom are shown.

For the 155 MeV beam, the DFW value of the MC calculations when passing through the Voronoi lung phantom and tough lung phantom were 0.39 and 0.24 cm, respectively.

For the 200 MeV beam, the DFW value of the MC calculations when passing through the Voronoi lung phantom and tough lung phantom were 0.48 and 0.36 cm, respectively. The results for the DFW value are shown in Table 2.

**Table 2:** Distal falloff widths (DFW) results for tough lung phantom and treatment planning system (TPS).

Method	Setup	155 MeV	200 MeV
		DFW (cm)	DFW (cm)
MC simulation	Pristine peak with tough lung phantom	0.24	0.36
MC simulation	SOBP30 with Voronoi lung phantom	0.46	0.61
TPS	SOBP30 with Voronoi lung phantom	0.29	0.45

### 3.4 Comparison with TPS

**Figure 7:**
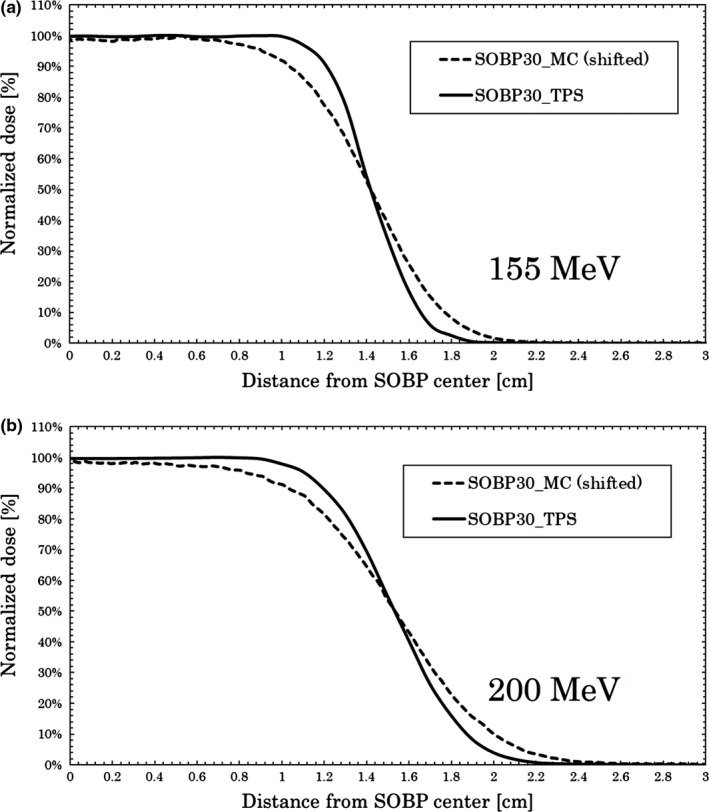
Monte Carlo (MC) and treatment planning system calculations for depth dose. Results at 155 MeV (a) and 200 MeV (b) for SOBP 30 mm with the Voronoi lung phantom. MC results were shifted to intersect at 50% to compare distal falloff widths.

Figure 7 shows the results of MC and TPS calculations for 155 MeV (a) and 200 MeV (b) when passing through the Voronoi lung phantom. The vertical axis of the graph was normalized by the maximum dose. The horizontal axis is the length in the depth direction from the center of SOBP (TPS).

For the 155 MeV beam, the DFW values of the MC and TPS calculations were 0.46 and 0.29 cm, respectively.

For the 200 MeV beam, the DFW values of the MC and TPS calculations were 0.61 and 0.45 cm, respectively. The results for the DFW value are shown in Table 2.

## 4 DISCUSSION

### 4.1 Comparison between measurements and MC calculations

The measurements and MC calculations were both sufficiently consistent in comparison to the results from the previous study.^7^

We compared the results obtained in this study to those obtained using an independent theoretical formula to investigate the validity of this experiment.

Assuming that the Bragg peak widening in the 80–20% portion, which defines DFW, follows a Gaussian distribution (σ), it becomes

(1) DFW≒1.13σ

where, using the formula above, σ_pristine_ ＝ 2.12 mm for the pristine peak and σ_Voronoi_ = 3.53 mm for the Voronoi lung phantom at 155 MeV, and σ_pristine_ ＝ 3.13 mm for the pristine peak and σ_Voronoi_ = 4.24 mm for the Voronoi lung phantom at 200 MeV.

In addition, σ_hetero_, the effect of introducing a heterogeneous material, can be determined from the following equation:

(2) $$\sigma_{\text{Voronoi}}^{2}=\sigma_{\text{pristine}}^{2}+\sigma_{\text{hetero}}^{2}$$

Ultimately, the effect of heterogeneity σ_hetero_ was σ_hetero_ = 2.82 mm for 155 MeV and σ_hetero_ = 2.86 mm for 200 MeV.

Additionally, from the theoretical formula in the literature,^7^ σ^2^(*z*) is given by

(3) $$\sigma^{2}\left(z\right)=\left(p-p^{2}\right)z\Delta$$

where p is determined as the average density of the Voronoi lung phantom (i.e., *p* = 0.237 g/cm^3^ for our phantom), z is the diameter of the phantom (i.e., *z* = 4 cm for our phantom), and Δ is the size of the structure that makes up the phantom.

The diameter of the branches of the Voronoi lung phantom was designed to be 0.4–0.8 mm, but some areas where the elements connect were as large as 1.6 mm in diameter.

Considering this, the difference between the minimum and maximum diameter becomes Δ = 0.4 to 1.6 mm.

When Δ = 0.4 mm, σ = 1.70 mm, and when Δ = 1.6 mm, σ = 3.40 mm, so the fact that the calculated results obtained in this study, σ_hetero_ = 2.82 mm (for 155 MeV) and σ_hetero_ = 2.86 mm (for 200 MeV) fall within this range indicates that they are consistent with the theoretical formula. This confirms that the experimental results obtained in this study are reasonable. The phantom fabricated in this study using the Voronoi tessellation has a 3D‐printable structure, and it behaves similarly to human lungs, which makes it useful for simulations.

### 4.2 Results of tough lung phantom and TPS

The conventional tough lung phantom, whose density is only consistent with the lungs, could not be used to calculate the effects of the heterogeneous structure. In the condition close to the clinic, SOBP was calculated with MC and TPS to verify the influence of the heterogeneous structure. There was a difference between DFW in MC and TPS, the main causes being the difference in the calculation algorithm and the influence of the voxel size of heterogeneous structure. According to the literature,^7^ it is known that the smaller the voxel size, the closer the DFW values to those of plastinated lung phantom. Since the SOBP is created by overlapping pristine peaks, it has more influence than pristine peaks. The uncertainty of the dose for the heterogeneous structure of this study shows that it is necessary to cope with the correction formula. From these results, it can be said that the phantom designed in this study is optimal to verify the influence of the heterogeneous structure.

It should be noted that this study did not address lung movement. However, by using a stretchable material and the shape of the Voronoi lung phantom, it is possible to measure the influence of lung motion.^19^ In future work, we plan to simulate lung motion by changing the stretchable material. Such investigations may be able to contribute toward improvement of the precision of measurements and calculations in radiation therapy.

## 5 CONCLUSIONS

In this study, we developed a new lung phantom for measurements and MC calculations by using Voronoi tessellation. In comparison to a plastinated lung sample, the designed Voronoi lung phantom yielded similar measured and calculated values. The results at 155 MeV were sufficiently consistent with the measured and calculated values for DFW. The peak values were also consistent with the measured and calculated values, within a maximum 5% error. The results at 200 MeV were also sufficiently consistent with the measured and calculated values for DFW, and the peak values were also consistent with the measured and calculated values, within a maximum 3% error. These results suggest that the 3D‐printable Voronoi lung phantom fabricated in this study is useful shape for simulating human lung.

## CONFLICT OF INTEREST

The authors declare no conflict of interest.
